# Sequence-based prediction of physicochemical interactions at protein functional sites using a function-and-interaction-annotated domain profile database

**DOI:** 10.1186/s12859-018-2206-2

**Published:** 2018-06-01

**Authors:** Min Han, Yifan Song, Jiaqiang Qian, Dengming Ming

**Affiliations:** 1 Department of Physiology and Biophysics, School of Life Science, Fudan University, Shanghai, 200438 People’s Republic of China; 2 College of Biotechnology and Pharmaceutical Engineering, Nanjing Tech University, Biotech Building Room B1-404, 30 South Puzhu Road, Jiangsu 211816 Nanjing, People’s Republic of China

**Keywords:** Physicochemical interaction prediction, Protein functional site prediction, *fi*DPD, Hidden Markov model, Domain profile module

## Abstract

**Background:**

Identifying protein functional sites (PFSs) and, particularly, the physicochemical interactions at these sites is critical to understanding protein functions and the biochemical reactions involved. Several knowledge-based methods have been developed for the prediction of PFSs; however, accurate methods for predicting the physicochemical interactions associated with PFSs are still lacking.

**Results:**

In this paper, we present a sequence-based method for the prediction of physicochemical interactions at PFSs. The method is based on a functional site and physicochemical interaction-annotated domain profile database, called *fi*DPD, which was built using protein domains found in the Protein Data Bank. This method was applied to 13 target proteins from the very recent Critical Assessment of Structure Prediction (CASP10/11), and our calculations gave a Matthews correlation coefficient (MCC) value of 0.66 for PFS prediction and an 80% recall in the prediction of the associated physicochemical interactions.

**Conclusions:**

Our results show that, in addition to the PFSs, the physical interactions at these sites are also conserved in the evolution of proteins. This work provides a valuable sequence-based tool for rational drug design and side-effect assessment. The method is freely available and can be accessed at http://202.119.249.49.

## Background

Most proteins perform biological functions via interactions with their partners, such as small molecules or ligands, DNA/RNA, and other proteins, forming instantaneous or permanent complex structures. Of particular importance is that only a few pivotal amino acids on a protein’s surface, usually called protein functional sites (PFSs), play key roles in determining these interactions. Thus, understanding protein functions depends upon accurate predictions of PFSs. However, PFSs alone do not reveal the details of their physicochemical interactions, which is indispensable information for understanding protein biochemical reactions. Together with PFS prediction, accurate protein-ligand interaction (PLI) prediction opens up a new dimension in correctly annotating protein function and thus provides valuable information for rational drug design and drug side-effect assessment [1–3]. To date, 3D protein-partner complex structures have been the main source of knowledge about PFSs and PLIs. In recent years, in silico methods have received increasing attention as an alternative strategy for protein function annotation, especially in predicting PFSs. The advantage of these methods stems from two factors: the rapid accumulation of a large number of complex 3D structures in publicly accessible databases such as the Protein Data Bank (PDB) [4] and the rapid development of computer technology and computation algorithms.

In the last few decades, many computational methods have emerged to identify PFSs from protein structures and sequences [5]. Most sequence-based methods assume that functionally important residues are conserved through evolution and can be identified as conserved sites based on multiple sequence alignment (MSA) within homologous protein families [6–8]. Sequence-based information such as secondary structure propensity and the likely solvent accessible surface area (SASA) have also been used to improve the prediction [9–12]. In addition, structure-based methods that essentially determine local or overall structural similarity have been developed for PFS prediction [13–16]. Typical local structural features include large clefts on protein surfaces [17, 18], special spatial arrangements of catalytic residues [19–21], and particular patterns between surface residues [22, 23]. Other prediction methods have used both structural and sequence information [24, 25] and might, when combined with artificial intelligence techniques, provide encouraging results [26–28]. Other methods based on protein dynamics [29–34], conventional molecular dynamics and docking simulations [35–37] have also been successful in PSF prediction. To elucidate the physicochemical interactions between proteins and their partners, particularly those between protein and ligands, researchers have attempted to characterize these interactions as early as the emergence of the first protein-ligand complex structure. However, only very recently have structural bioinformatic tools emerged with which to systematically characterize protein-ligand interactions (PLIs) [38–43] due to the rapid accumulation of protein complex structures. Additionally, a few databases record detailed atomic interactions between proteins and ligands, facilitating PLI studies [44–46]. These data provide new resources for the large-scale characterization of physicochemical interactions between proteins and their partners and have helped improve conventional docking simulation and pharmacology research. Several knowledge-based or ab initio methods have been developed for the prediction of PFSs; however, an accurate method for predicting the physicochemical interactions associated with PFSs is still lacking [47].

In this paper, we develop a new method for predicting physical interactions occurring on functional sites based on the amino acid sequences of given proteins. This sequence-based method first predicts PFSs from a functional site-annotated domain profile database, or *f*DPD, and then assigns the types of interactions most likely to appear at the predicted sites. In this study, we derived a functional site- and interaction-annotated domain profile database, called *fi*DPD, which plays the primary role in the prediction. A profile hidden Markov model of the HMMER program was used in the prediction to search a module member of the database for a given protein. We applied the *fi*DPD method to 10 target proteins of CASP10 [48] and CASP11 [49] and found that the method has a Matthews correlation coefficient (MCC) value of 0.66 for PFS prediction. Additionally, the model provided a correct physicochemical interaction prediction for 80% of the examined sites. We expect the present method to be a valuable auxiliary tool for conventional bioinformatic and protein function annotations.

## Methods

**Fig. 1:**
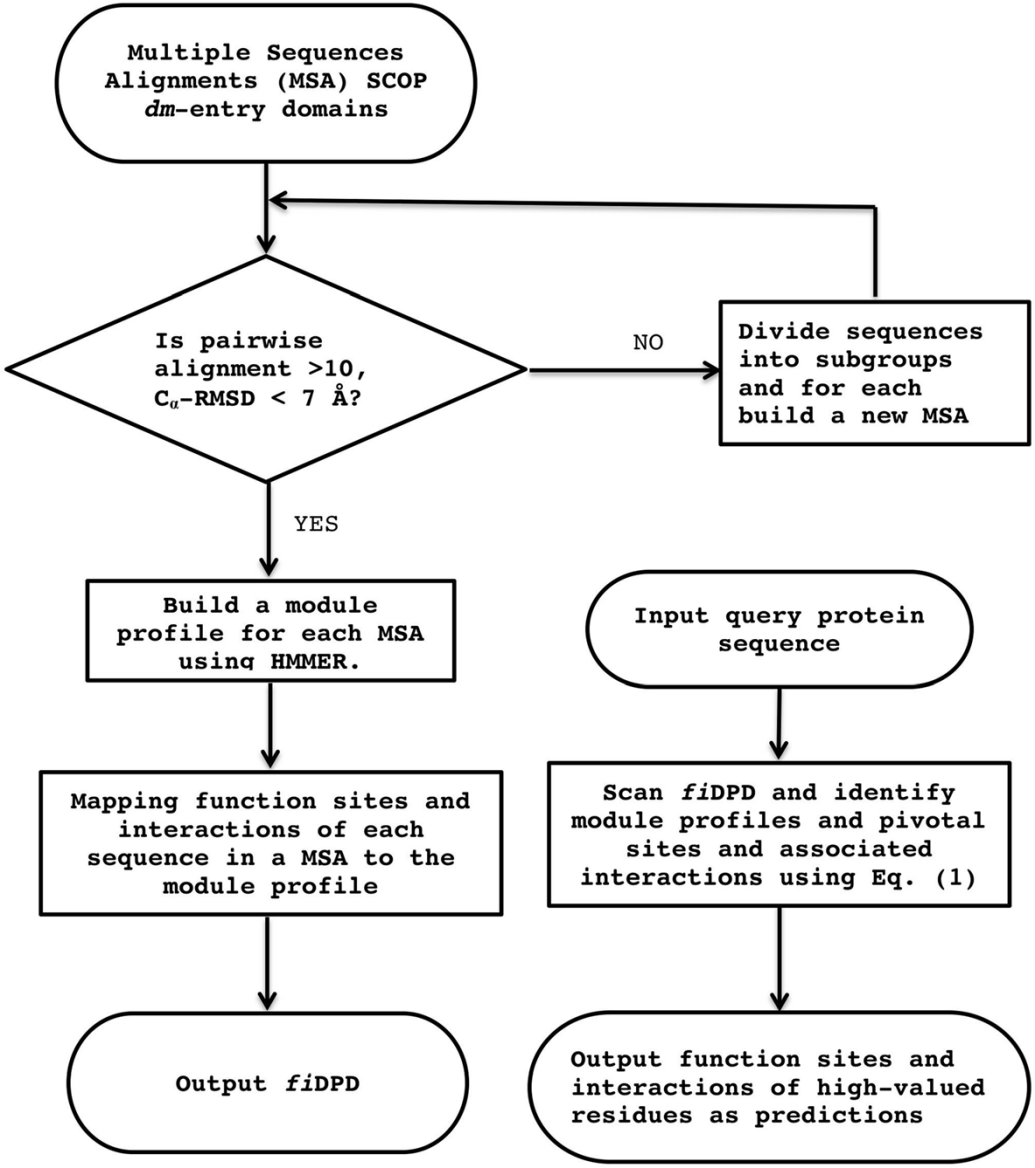
Flow-chart for building the function-site- and interaction-annotated domain profile database (fiDPD) and for predicting protein function-sites and PLIs using fiDSPD

Figure 1 shows the flow chart used to build *fi*DPD. We first introduced the *f*DPD as a list of representative profile modules built by sorting out structure-and-sequence similar protein domains in the SCOP databases [50]. Next, PFSs and atomic patterns of PLIs were derived from known protein-ligand-complex structures in the PDB; then, after a series of site-to-site mappings, these structures were used to annotate *f*DPD profile modules and thus to build the *fi*DPD.

### *f*DPD was prepared based on the subgroup classification of domain entries of the SCOP database

We started with a modified classification of protein domain structures collected in the SCOP database [50, 51]. In SCOP, a large protein structure is often manually divided into a few smaller parts or domains according to their spatial arrangement within the protein. A recent version of SCOPe 2.05 was downloaded from http://scop.berkeley.edu/references/ver=2.05, which includes 214,547 domain entries extracted from 75,226 protein structures in the PDB. In SCOP, these domain structures are arranged in a hierarchical 7-level system—Class (*cl*), Fold (cf), Superfamily (*sf*), Family (*fa*), Protein Domain (*dm*), Species (*sp*), and PDB code identity (*px*)—according to their sequence, function and structure similarity. Specifically, those domains listed in a given domain entry (*dm*) presumably share the same class, fold, superfamily and protein family but might differ in species and PDB code entry. Theoretically, PFSs are more likely to be conserved when they share both higher structural and sequential similarity, and this assumption forms the basis for our algorithm of *fi*DPD in the prediction of PFSs and PLIs. Using a profile hidden Markov model of the HMMER program, the MSA of all the domains within the same *dm* entry gives a single representative profile module. In this way, 12,527 representative profile modules were created for all the *dm* entries, forming the basis of *f*DPD and *fi*DPD.

In building *f*DPD, it is important for protein domains within the same *dm* entry to be structurally and sequentially close to one another. However, a quick calculation reveals that the *C*_α_ root-mean-square-distance (RMSD) can be as large as 12 Å for many domain structures listed in the same *dm* entry. This result indicates that there are many domains listed in the same *dm* entry of SCOPe 2.05 that have quite different structures, which makes the profile modules of *f*DPD less representative of member proteins within the *dm* entry. To reduce the difference, we divided the domains within a *dm* entry into a few smaller groups or subgroups so that selected domains within the same subgroup would have mutual *C*_α_-RMSD < 7 Å and a mutual sequence similarity > 10 (a score calculated by the MSA program CLUSTALW [52]). Thus, derived subgroups then replace the *dm* entry as the basic unit of *f*DPD. *f*DPD contains 16,559 subgroups, which is 32% more than the original SCOP *dm* entries, with approximately 12 member structures in each subgroup, on average.

### *f*DPD is composed of functional site annotated protein profile modules based on multiple subgroup-protein sequence alignment

In *f*DPD, sequences of protein domains in a subgroup were extracted and aligned using the MSA program MUSCLE [53], from which a profile module was then built using the *hmmbuild* module of the HMMER program (http://hmmer.org/ [54]). A profile module is a sequence of hypothetical amino acids, which is, instead of conventional amino acids, probably a mixture of certain amino acids according to the MSA of the subgroup. For each individual position in a profile module, we defined a conservation value *C* according to the MSA. We assigned the *C* value as 0, 1, 3, or 4 for a position being nonconservative, minimally conservative, conservative and highly conservative, as indicated respectively by a gap, “+” symbol, a lowercase letter or a capital letter in the MUSCLE alignment. We also defined an overall volume value *N* for a profile module as the number of protein domains listed in the subgroup: a larger *N* value usually indicates that more information is available for that subgroup and thus a greater confidence on the annotation.

**Fig. 2:**
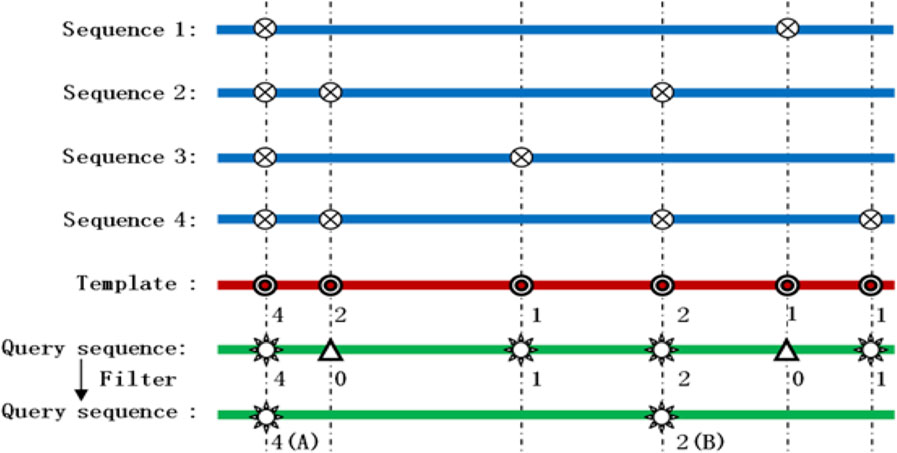
Mapping known protein function sites and interactions to a domain-profile module, ⊗: known PFSs of domain structures, ⊙: pivotal PFSs in a profile module with the number indicating a weight factor, *: PFSs mapped into the query protein sequence from profile module pivotal sites, which, after a filtering, is reduced to two points (A and B) as a final prediction output, Δ: non-conservative pivotal sites mapped into the query protein, which will be ignored due to the low conservation value

A scoring function *S* was assigned to each position in an *f*DPD profile module to mark its propensity of being a functional site. To this end, we first mapped known functional sites of member proteins within the same subgroup to the profile module according to the MSA (see Fig. 2). Functional sites of member proteins were collected from the SITE sections of the corresponding PDB file. Of the 202,705 protein domains listed in SCOPe, 132,725 domain structures have a total of 1,878,004 functional sites annotated in PDB SITE records. Then, for simplicity, we assigned *S* as the total hit number that a profile module position received based on the MSA. Thus, the larger a position’s *S*-value, the more likely it is to be a hypothetical functional site for the profile module. In this way, the profile modules were annotated with known PFSs, and we called the database composed of these profile modules the *function-site*-annotated domain profile database, or *f*DPD. Previously, alternative functional site annotations for profile modules were also built by using different “known” PFSs derived from FDPA calculations instead of those recorded active sites in the PDB database [55]. Compared with the *dm* entries in the original SCOP, in *f*DPD, PFSs should be more likely to be conserved since they share both higher structural and higher sequential similarity.

### *fi*DPD was built by attaching physicochemical interaction annotations to functional sites in *f*DPD profile modules

Obviously, the abovementioned *S*-value is heavily dependent on the means by which the “known” PFSs were determined. In this work, *S*-values are determined by using only PDB SITE information, which, in most cases, is composed of manually prepared ligand-binding sites. Other types of biologically relevant functional site data, such as enzyme active sites [56] and phosphorylation sites [57], might also be used in the annotation. Here, considering the importance of PLIs in determining protein function, we added PLI annotations to the profile modules of *f*DPD to build the *function-site* and interaction-annotated domain profile database, or *fi*DPD.

To annotate the profile modules with PLIs, atomic interaction patterns between the protein and ligand were initially determined based on their 3D protein-ligand complex structures. Specifically, the atomic 3D coordinates of amino acids listed in PDB SITE sections and those of ligand molecules were filtered out from the PDB files; then, a series of atomic distances (*d*) were calculated between PFSs (A_Site_) and ligands (A_Ligand_). Finally, a few types of bonding and nonbonding interactions for each A_Site_ were determined based on the pairwise distances and the biochemical properties of involved amino acids.

#### H-bond

Almost all PLIs occur in aqueous environments, where water molecules play a critical role. As a result, hydrogen bonds might be consistently established and destroyed until a certain stable protein-ligand configuration is achieved. Here, we have calculated hydrogen bonds within the protein-ligand complex using the program HBPLUS [58]. The program determines H-bond donor (D) and acceptor (A) atom pairs based on a nonhydrogen atom configuration using a maximum H–A distance of 2.5 Å, a maximum D–A distance of 3.9 Å, a minimum D–H–A angle of 90° and a minimum H–A–AA angle of 90°, where H is the theoretical hydrogen atom and AA is the atom of functional sites in the H-bond acceptor. In this way, we defined NHBA and NHBD as the total number of H-bond acceptors and H-bond donors, respectively, associated with atoms in a given functional site.

#### Electrostatic interactions

Electrostatic force plays important roles in many PLIs and might be the main driving force to initiate catalytic reactions, to guide the recognition between protein and ligand, and so on [59–61]. However, accurately determining atomic charges in bio-structure is a very challenging task since it is highly sensitive to the surrounding environment. Here, for simplicity, we identified electrostatic interactions simply by examining the charging status of contact atoms in PLIs. Specifically, we first selected positively charged nitrogen (N) atoms of functional sites of Arg, His, and Lys and then determined an electrostatic interaction if there a neighboring (< 4.5 Å) oxygen atom was present in the ligand, which is not part of a cyclized structure. An electrostatic interaction was also built when a negatively charged oxygen (O) atom from Asp and Glu residues was found near a ligand nitrogen atom. We used NELE as the total number of electrostatic interactions involving atoms in a given functional site.

#### π-stacking interactions

π-Stacking interactions play a critical role in orientating ligands inside binding pockets. We first identified the aromatic side chains of Trp, Phe, Tyr and His of PFSs and carbon-dominant cyclized structures of ligands. Usually, aromatic rings form an effective π-stacking interaction when they get close enough (4.5–7 Å) and have either a parallel or perpendicular orientation [62, 63]. Here, for simplicity, we defined a π-stacking interaction if we could find three or more distinct heavy-atom pairs between atoms from the aromatic ring of a given functional site and those from ligand carbon-ring structures. We defined the total number of π-stacking interactions involving a given functional site as NPI.

#### Van der Waals interaction

A Van der Waals interaction is formed when the distance *d* between a nonhydrogen atom of protein functional site and a nonhydrogen atom of ligands satisfies the following inequality:

$$ d<\mathrm{vdW}\left({A}_{\mathrm{Site}}\right)+\mathrm{vdW}\left({A}_{\mathrm{Ligand}}\right)+0.5\ \mathrm{\AA}, $$

where vdW(*A*) is the Van der Walls radius of atom A and no covalent bond, coordination bond, hydrogen bond, electrostatic force or π-stacking interaction is found between them. A similar definition of the Van der Waals interaction was also used by Kurgan and colleagues in their study of protein-small ligand interaction patterns [38] and by Ma and colleagues in their study of protein-protein interactions [64]. The atomic Van der Waals radii were taken from the CHARMM22 force field [65]. Each functional site was assigned an NVDW value as the total number of Van der Waals interactions involving atoms of this site.

#### Covalent bond and coordinate bond

Usually, nonbonded forces dominate interactions between a ligand and its target protein; however, irreversible covalent bonds are also found in PLIs when a tight and steady connection between the ligand and receptor is essential to the biological function, such as in the rhodopsin system [66]. A covalent bond is formed if the distance between a nonhydrogen atom from a functional site and a nonhydrogen atom from ligand satisfies $d<R\left({\mathrm{A}}_{\mathrm{Site}}\right)+R\left({\mathrm{A}}_{\mathrm{Ligand}}\right)+0.5\ \mathrm{\AA}$, where *R*(A) is the radius of atom A. For metal-ion ligands, this condition also defines coordinate bonds between metal ions and PFSs. Usually, in coordinate bonds, the shared electrons are present in atoms with higher electronegativity in a functional site. We denoted NCOV as the total number of covalent bonds involving atoms in the functional site and NCOO as the total number of coordinate bonds involving atoms in that site.

We characterized a PLI between a PFS and the ligands with a 7-dimensional interaction vector **V** = (NCOV, NCOO, NHBA, NHBD, NPI, NELE, NVDW). The interaction vectors of all member proteins were summed in different pivotal sites of the profile module according to the MSA of the studied subgroup. As a result, each *f*DPD profile module was annotated with interaction vectors **V** on hypothetical functional sites, thus forming the *fi*DPD.

### *fi*DPD predicts both functional sites and PLIs using a hidden Markov model

*fi*DPD is essentially a list of profile module entries annotated with domain functional sites and PLIs. In *fi*DPD, two steps are required to predict the hypothetical functional sites and involved PLIs for a given inquiry protein: 1) identifying profile modules in *fi*DPD that match the query sequence best and 2) interpreting pivotal functional sites and associated PLIs of the matched profile modules as a prediction of PFSs and PLIs for the query protein based on certain statistical evaluations.

In the first step, *fi*DPD scans the query sequence against all its module entries using the SCAN module of the HMMER program [67]. The scan usually gives a couple of profile modules within an alignment E-value cutoff no greater than 1 × 10^− 5^. Each alignment (indexed by superscript *j* in Eq. (1)) is assigned a scoring function *E* as the negative logarithm of the E-value score. Due to the limited volume of known protein sequences contained in *fi*DPD, there are cases in which HMMER SCAN cannot find any match for the query protein, and for these cases, *fi*DPD simply gives a notice of “no-hit.” In step 2), we defined a scoring function *F*_*i*_ for the *i*th residue of the query protein as its propensity to be a functional site:

1 $$ {F}_i={\sum}_j{S}_{i^{\prime}}^j{C}_{i^{\prime}}^j{N}^j{E}^j $$

where the summation runs over all the alignments *j* and *i*^′^ stands for the position of the profile module that matches the *i*th residue of the query protein. Residues with a high*-*valued *F*-scoring function will be predicted as hypothetical functional sites.

One way to determine high-*F*-valued sites for a query protein is to simply choose a certain number (*n*) of top-valued residues, called *n*-top selection. This method has been used for enzyme catalytic site prediction [55] since experimentally determined enzyme active sites have a relatively fixed number as revealed by the Catalytic Site Atlas (CSA) dataset [56]. Another method to select top-valued residues uses a cutoff percentage that was proved to be efficient in a previous ligand-binding site prediction study [32, 34]. In this method, we first filtered out those low-valued noise-like residues whose *F****-***scores were smaller than a cutoff percentage *M*% of the maximum *F*-value *F*_*max*_; then, for the remaining residues, the top *T*% were predicted as hypothetical functional sites of the query protein. Usually, this selection strategy tends to give a greater prediction function for larger proteins. We used this selection strategy to predict PFSs in the remainder of this paper. The server is freely available and can be accessed at http://202.119.249.49. For clarity, *F****-***scores are renormalized to a 1–100 range for predicted sites.

To predict PLIs, we defined a protein-ligand interaction scoring-vector function **I**_*i*_ = {NCOV_*i*_, NCOO_*i*_, NHBA_*i*_, NHBD_*i*_, NPI_*i*_, NELE_*i*_, NVDW_*i*_} for the *i*th residue of the query protein following Eq. (1):

2 $$ {\boldsymbol{I}}_i={\sum}_j{N}^j{E}^j{C}_{i^{\prime}}^j{\mathbf{V}}_{i^{\prime}}^j $$

where ${\mathbf{V}}_{i^{\prime}}^j=\left\{{\mathrm{NCOV}}_{i^{\prime}}^j,{\mathrm{NCOO}}_{i^{\prime}}^j,{\mathrm{NHBA}}_{i^{\prime}}^j,{\mathrm{NHBD}}_{i^{\prime}}^j,{\mathrm{NPI}}_{i^{\prime}}^j,{\mathrm{NELE}}_{i^{\prime}}^j,{\mathrm{NVDW}}_{i^{\prime}}^j\right\}$ is the PLI vector for residue *i*^′^ in the profile module *j* that matches the *i*th residue of the query sequence. For each prediction functional site, *fi*DPD will determine an associated PLI vector according to Eq. (2), which identifies the interactions involved with each prediction site. For clarity, in the webserver, when ***I***_*i*_ has a nonzero value from Eq. (2), it will be simply assigned as “1” to indicate a certain type of PLI.

#### Validation datasets

The original *f*DPD was examined for PFS prediction using a few types of datasets, including two manually cultivated enzyme catalytic site datasets of the 140-enzyme CATRES-FAM [68], the 94-enzyme Catalytic Site Atlas (CSA-FAM) [56] and a 30-member small-molecular binding protein target from CSAP9 [69]. Here, we examined *fi*DPD by calculating the PLIs of protein targets listed in CASP10 [70] and in CASP11 [49], whose ligand-binding complex structures had been solved.

#### Validation method

The conventional prediction precision and recall calculations were used to evaluate the performance of our method: Precision = TP/(TP + FP) and Recall = TP/(TP + FN), where the true positives (TPs) are the predicted residues listed as functional sites in the dataset, the false positives (FPs) are the predicted sites not listed in the dataset, and the false negatives (FNs) are the functional sites listed in the dataset but missed by the method. Another relevant quantity is the true negative (TN), which stands for the correctly predicted nonbinding/nonfunctional site residues. In our calculations, the statistics did not take account of the “no-hit” predictions. The overall precision is the sum of all the TPs divided by the total number of predicted residues, and the overall recall is the sum of all the TPs divided by the total number of listed functional sites in the dataset. The precision-recall curve was found to be slightly dependent on the cutoff percentage M% and T% in the selection method. The MCC [71] was used to assess the ligand-binding residue predictions of the CASP10 target proteins [72] and is defined as follows:

$$ \mathrm{MCC}=\frac{\mathrm{TP}\times \mathrm{TN}-\mathrm{FP}\times \mathrm{FN}}{\sqrt{\left(\mathrm{TP}+\mathrm{FP}\right)\bullet \left(\mathrm{TP}+\mathrm{FN}\right)\bullet \left(\mathrm{TN}+\mathrm{FP}\right)\bullet \left(\mathrm{TN}+\mathrm{FN}\right)}}. $$

The predicted PLIs were compared with those directly derived from 3D protein-ligand complex structures, and precision and recall values were obtained to qualify PLI predictions.

## Results and discussion

### The mimivirus sulfhydryl oxidase R596

**Fig. 3:**
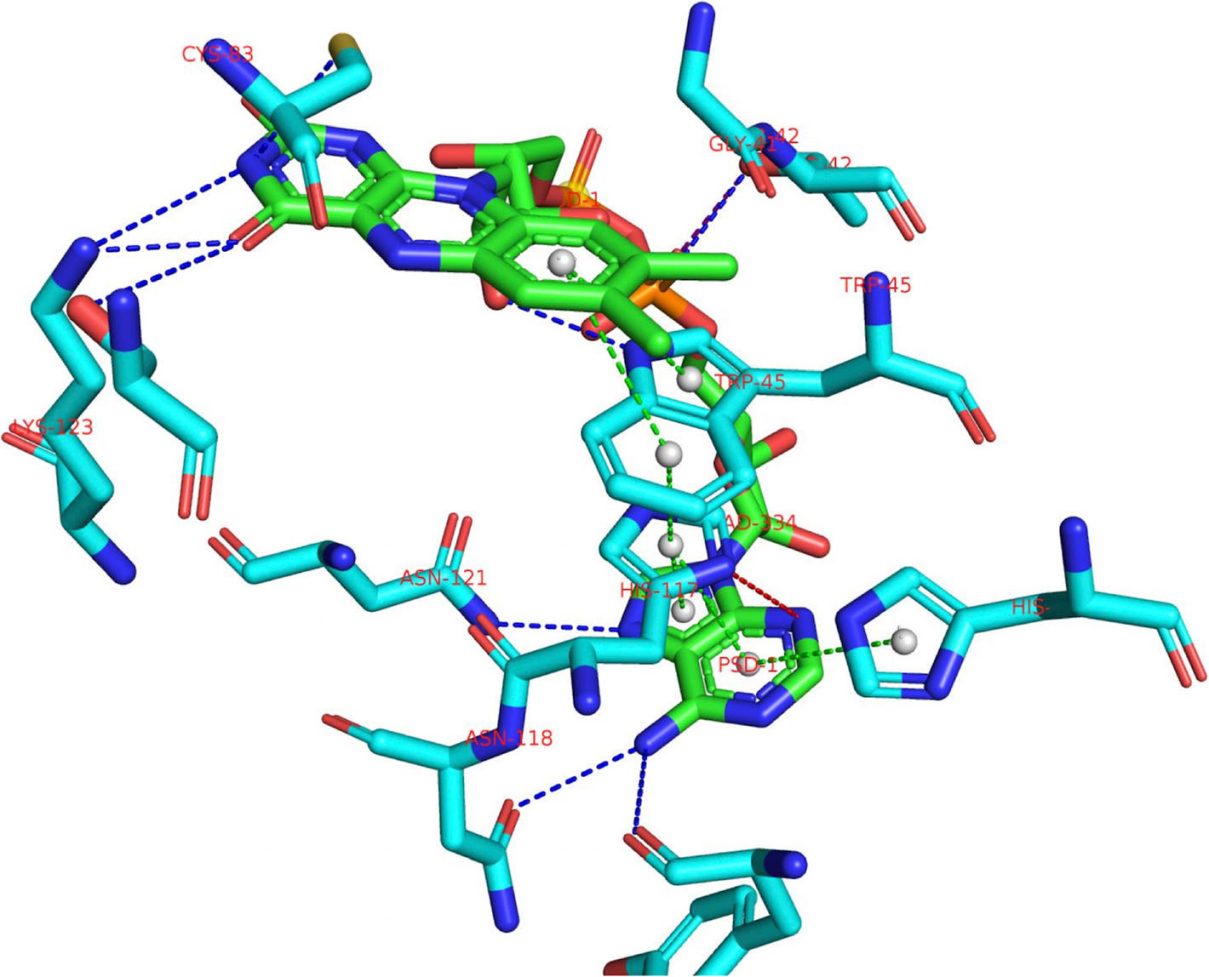
Mapping the protein-ligand interactions predicted for the mimivirus sulfhydryl oxidase R596, target T0737, PDB code 3TD7. Dash lines represent PLIs, they are colored as following: blue for electrostatic interactions, green for π-stacking interactions, gray for van der Waals interactions, and red for interaction not found by fiDPD

The 292aa mimivirus sulfhydryl oxidase R596 is target T0737 of CASP10, whose structure was later determined at 2.21 Å (PDB entry 3TD7; see Fig. 3 [73]). The protein is composed of two all alpha-helix domains: the N-terminal sulfhydryl oxidase domain (Erv domain) and the C-terminal ORFan domain. The mimivirus enzyme R596 has an EC number of EC1.8.3.2, catalyzing the formation of disulfide bonds through an oxidation reaction with the help of a cofactor of flavin adenine dinucleotide (FAD). FAD is tightly bonded to 22 residues in the catalytic pocket in the Erv domain [48], playing an important role in transferring electrons from a 10 Å distance shuttle disulfide in the flexible interdomain loop to the active-site disulfide close to FAD in the Erv domain [73]. In the prediction, *fi*DPD scanned the T0737 sequence against the database and found 4 profile module entries, all from the Apolipoprotein family with a structure of a four-helical up-and-down bundle. The 4 entries include an automated-match-domain profile built from 10 sequences from *Arabidopsis thaliana*, a second automated-match-domain profile built from 4 sequences from *Rattus norvegicus*, an augmenter of liver regeneration domain profile built from 13 sequences from *Rattus norvegicus*, and a thiol-oxidase Erv2p domain profile built from 6 sequences from *Saccharomyces cerevisiae*. The scanning E-value ranges from 2 × 10^− 8^ to 1 × 10^− 19^, indicating that the query sequence only has moderate similarity with the annotated sequences in the database. A total of 56 annotated pivotal sites in the 4 *fi*DPD profile modules were then collected and sorted according to their functional site scoring functions. When mapping to the query sequence, 12 functional sites were then automatically identified, resulting in a 92% prediction precision and 57% recall. We also examined those functional sites that *fi*DPD failed to identify and found that they are located in a different C-terminal domain than the four-helical up-and-down bundle domain.

To examine the PLI prediction, we first collected interaction scoring vectors associated with pivotal sites in the four profile modules according Eq. (2) and then compared with those directly determined from the protein-ligand complex structure recorded in PDB entry 3TD7 (Table 1). Figure 3 demonstrates key interactions predicted by Eq. (2) and those not found by the prediction. *fi*DPD correctly predicted all the π-stacking interactions involving Trp45, His49, Tyr114, and His117, indicating that π-π interactions play a critically important role in ligand binding. The prediction also found significant π-stacking interactions on pivotal sites of Leu78 and Lys123; however, these π-π interaction predictions were ignored in posttreatment simply because of the lack of aromatic side chains in these residues. *fi*DPD also found the correct electrostatic interactions on His117 and Lys123 sites. The algorithm identified a large probability of electrostatic interactions on sites Thr42 and Val126; however, these interactions were ignored in posttreatment since the involved residues are not chargeable in the conventional conditions. In total, approximately 80% of the overall PLI predictions were associated with identified functional sites.

**Table 1:** The prediction of protein-ligand interactions on PFSs of T0737†

Target	Site	AA	COV	COO	ELE	HBD	HBA	π-π
T0737	41	G	0	0	0	0	0	0
	42	T	0	0	+/0	T	0	0
	45	W	0	0	0	T	0	T
	49	H	0	0	0	0	+	T
	78	L	0	0	0	0	0	0
	83	C	0	0	0	+	T	0
	114	Y	0	0	0	0	T	T
	117	H	0	0	T	+	–	T
	118	N	0	0	0	+	T	0
	120	V	0	0	0	0	0	0
	121	N	0	0	0	0	+	0
	123	K	0	0	T	T	+	+/0

†AA stands for amino acid, COV for covalent bond, COO for coordinate bond, ELE for electrostatic interaction, HBD for H-bond donor, HBA for H-bond acceptor, π-π for π-stacking interactions. “0” indicates the corresponding interaction is not present in protein-ligand complex structure and *fi*DPD calculation also showed no such type PLIs on the site

### CASP10 and CASP11 targets

We applied *fi*DPD to protein targets listed in CASP10 and CASP11, of which 13 targets had been solved with explicit bound ligands [48]. Table 2 lists all the predictions, of which *fi*DPD gave a no-hit for 3 target proteins. For the remaining 10 predictions, *fi*DPD gave an overall precision of 64% and an overall recall of 46% using a scale selection with T of 45% and M of 35%. The averaged MCC of the predictions was 0.49. Considering the ligand-binding types, we found that *fi*DPD provided better functional site predictions for metal binding sites with an average MCC value of 0.68, while it was 0.38 for nonmetal binding site prediction, indicating that PFSs are more conservative with respect to either spatial arrangement or sequence location in metal binding.

**Table 2:** Ligand-binding sites predictions of CASP10/11 targets proteins†

Target	PDB	Ligand	Type	Sites*	Prediction	TP	Precision	Recall	MCC
T0652	4HG0	AMP	Non-metal	11	17	6	0.35	0.55	0.41
T0657	2LUL	ZN	Metal	5	9	4	0.44	0.8	0.58
T0659	4ESN	ZN	Metal	3	No-hit				
T0675	2LV2	ZN	Metal	8	9	8	0.89	1	0.94
T0686	4HQL	MG	Metal	5	6	3	0.5	0.6	0.54
T0696	4RT5	NA	Metal	6	3	1	0.33	0.17	0.21
T0697	4RIT	TRS	Non-metal	6	11	0	0	0	0
T0706	4RCK	MG	Metal	5	3	3	1	0.6	0.77
T0720	4IC1	MN/SF4	Metal	14	No-hit				
T0721	4FK1	FAD	Non-metal	29	3	3	1	0.1	0.31
T0726	4FGM	ZN	Metal	7	No-hit				
T0737	3TD7	FAD	Non-metal	21	13	12	0.92	0.57	0.71
T0744	2YMV	FNR	Non-metal	19	4	4	1	0.21	0.45

† Target 762 to 854 were taken from CASP11 whose protein-ligand interactions were well characterized in the crystal structures

*“Sites” is the number of ligand-binding sites recorded in PDB files of the target protein

We compared the performance of *fi*DPD with the recently published ligand-binding site prediction methods LIBRA [74] (Table 3) and COACH [75, 76] (Table 4). LIBRA aligns the structures of input proteins with a collection of known functional sites and gives an averaged MCC of 0.57 for the studied target proteins. Six LIBRA predictions were based on the known sites of the PDB structures of the target proteins themselves and contributed a higher average MCC value of 0.80. For COACH, whose prediction is sequence based, the average MCC was 0.58, of which 2 predictions were based on the known sites of the target PDB structures. We observed that, except for T0675 and T0697, COACH had already used the target PDB structures as templates in building structures from input target protein sequences. Taken together, COACH performed best, while *fi*DPD’s performance (the present version of the database *fi*DPD does not contain target proteins except for T0675) was comparable with that of LIBRA, especially when known sites of the target PDB structures were not used.

**Table 3:** Prediction performance of LIBRA*

Target	PDB	Length	Sites	LIBRA Rank-1				LIBRA Rank-2			
				Prediction	TP	Model	MCC	Prediction	TP	Model	MCC
T0652	4HG0	292	11	7	1	N	0.08	8	7	N	0.74
T0657	2LUL	154	5	4	4	Y	0.89	4	0	N	0
T0659	4ESN	72	3	3	3	Y	1	3	0	N	0
T0675	2LV2	74	8	4	4	Y	0.69	4	4	N	0.69
T0686	4HQL	242	5	3	3	Y	0.77	3	3	Y	0.77
T0696	4RT5	111	6	7	0	N	0	5	0	N	0
T0697	4RIT	483	6	14	0	N	0	5	0	N	0
T0706	4RCK	217	5	3	0	N	0	8	1	N	0.14
T0720	4IC1	202	8	4	4	Y	0.7	5	0	N	0
T0721	4FK1	301	29	24	23	N	0.86	23	2	N	0.01
T0726	4FGM	589	7	6	6	N	0.92	10	0	N	0
T0737	3TD7	292	21	10	10	N	0.67	6	0	N	0
T0744	2YMV	329	19	12	12	Y	0.78	2	2	Y	0.64

*LIBRA prediction was based on the input of the PDBs of the target proteins. “Sites” is the number of ligand-binding sites recorded in PDB files of the target protein. “Y” in “Model” indicates that the prediction was made based on binding pockets in the PDB of the target protein as the template. “N” when the PDB of the target protein was not used in prediction

**Table 4:** Prediction performance of COACH*

Target	PDB	Length	Sites	COACH Rank-1				COACH Rank-2			
				Prediction	TP	Model	MCC	Prediction	TP	Model	MCC
T0652	4HG0	292	11	12	2	N	0.14	19	2	N	0.09
T0657	2LUL	154	5	7	0	N	0	5	5	Y	1
T0659	4ESN	72	3	3	3	N	1	8	0	N	0
T0675	2LV2	74	8	4	3	N	0.49	4	4	N	0.69
T0686	4HQL	242	5	4	3	N	0.66	13	0	N	0
T0696	4RT5	111	6	5	4	N	0.72	3	1	N	0.2
T0697	4RIT	483	6	12	0	N	0	5	0	N	0
T0706	4RCK	217	5	3	3	N	0.77	5	4	N	0.79
T0720	4IC1	202	8	5	4	Y	0.62	8	4	Y	0.48
T0721	4FK1	301	29	32	24	N	0.76	19	2	N	0.01
T0726	4FGM	589	7	10	6	N	0.71	10	3	N	0.35
T0737	3TD7	292	21	21	15	N	0.69	6	1	Y	0.05
T0744	2YMV	329	19	19	18	Y	0.94	7	4	N	0.32

*COACH built structures from the sequences of target proteins except for T0675 and T0697 by directly using the PDBs of the corresponding target proteins themselves. “Sites” is the number of ligand-binding sites recorded in PDB files of the target protein. “Y” in “Model” indicates that the prediction was made based on binding pockets in the PDB of the target protein as the template. “N” when the PDB of the target protein was not used in prediction

One of the key aspects of *fi*DPD predictions lies in the identification of physicochemical interactions between predicted binding sites and ligands. We examined the performance of the *fi*DPD prediction of PLIs in these target proteins by determining the overlap between the predicted PLIs and those calculated based on solved protein-ligand complex structures. Table 5 compared the predicted PLIs on functional sites with the experimental PLIs. In most cases, *fi*DPD can correctly identify 80% or more of the PLIs on functional sites.

**Table 5:** PLI predictions of CASP10/11 targets proteins†

Target	Interactions	Correct Prediction	Recall
T0652	60	36	60%
T0657	24	23	95.80%
T0675	30	28	93.30%
T0686	18	17	94.40%
T0696	18	15	83.30%
T0697	104	72	69.20%
T0706	24	21	87.50%
T0720	78	58	74.40%
T0721	60	50	83.30%
T0737	72	63	87.50%
T0744	42	37	88.10%
T0762	42	35	83.30%
T0764	60	52	86.70%
T0770	18	14	77.80%
T0784	18	18	100%
T0854	24	20	83.30%

† Target 762 to 854 were taken from CASP11 whose protein-ligand interactions were well characterized in the crystal structures

## Conclusions

In this paper, we present a new functional site- and physicochemical interaction-annotated domain profile database (*fi*DPD), from which we developed a sequence-based method for predicting both PFSs and PLIs. Our method is based on the assumption that proteins that share similar structure and sequence tend to have similar functional sites located on the same positions on a protein’s surface. A profile module entry in *fi*DPD is representative of a bunch of annotated domain structures that share high sequence and structure similarity. The *fi*DPD method first identifies profile modules in the database and then, as a prediction, maps the annotated pivotal sites and associated interactions of the module(s) to the residues of the query protein.

In a previous study, we examined the *f*DPD method with a collection of catalytic sites from a standard dataset of the 140-enzyme CATRES-FAM [68] and found that the method provided an enzyme active-site prediction of 59% recall at a precision of 18.3%. For ligand-binding site prediction of target proteins in CASP9, the method obtained an averaged MCC of 0.56, ranking between 8th and 10th of the 33 participating groups [72]. In this study, *fi*DPD gives new prediction for physicochemical interactions associated with the predicted PFSs. Here, *fi*DPD was applied to predict the functional sites of 10 target proteins in CASP10 and CASP11 that have been solved in a ligand-bound state and achieved an averaged MCC of 0.66. When compared with the solved 3D complex structures, we found that the predicted PLIs correctly overlapped 80% of the true PLIs. Our calculations indicate that the PLIs are well-conserved biochemical properties during protein evolution and that it is possible to assign accurate PLIs to predicted PFSs using an annotated database. *fi*DPD demonstrates that atomic physicochemical interactions between proteins and ligands can be reliably identified from protein sequences.

*fi*DPD is improvable. First, new annotations could be assigned to *fi*DPD to add new types of predictions. For example, adding annotations of enzyme catalytic sites (CSA), ligand-specific models, such as zinc-binding sites or RNA-binding sites, should endow *fi*DPD with the corresponding capability to predict catalytic sites, zinc-binding sites or RNA-binding sites. Annotations of *fi*DPD modules using other resources, such as dynamic simulations, FDPA calculations [32], pocket druggability [77], drug-target interactions (DTIs), drug modes of action [78], etc., should provide new content for *fi*DPD predictions that involve the protein dynamics and drug activity in PLIs. Second, considering that the classification of binding sites plays a key role in drug discovery and design, it would be interesting to use the clustering sites [79, 80] instead of the intact SITE information to annotate the database, which might make the prediction more useful. As a knowledge-based method, the utility and efficiency of *fi*DPD prediction suffers from the sampling limitation of annotations of known proteins. This sampling problem might be partially solved with large-scale protein sequencing efforts and worldwide structural genomics projects.

## Abbreviations

CASP: Critical Assessment of Structure Prediction
FDPA: Fast dynamics perturbation analysis
*fi*DPD: Function-site- and physicochemical interaction-annotated domain-profile-database
HMM: Hidden Markov Model
MCC: Matthews correlation coefficient
MSA: Multiple sequence alignment
PFS: Protein functional site
PLI: Protein-ligand interaction
RMSD: Root-mean-square-distance
SCOPe: Structural classification of proteins—extended
